# Nano-AgCu Alloy on Wood Surface for Mold Resistance

**DOI:** 10.3390/nano12071192

**Published:** 2022-04-02

**Authors:** Yanran Qi, Xiaohan Dai, Lianxiang Wei, Hongxue Luo, Yiliang Liu, Xiaoying Dong, Dequan Yang, Yongfeng Li

**Affiliations:** 1 State Forestry and Grassland Administration Key Laboratory of Silviculture in Down-Stream Areas of the Yellow River, College of Forestry, Shandong Agricultural University, Taian 271018, China; qyran1994@163.com (Y.Q.); dxiaohan0315@126.com (X.D.); 15662006529@163.com (L.W.); luohongxue1997@163.com (H.L.); liuyiliang0207@163.com (Y.L.); 2 Solmont Technology Wuxi Co., Ltd., 228 Linghu Blvd., Tian’an Tech Park, A1-602, Xinwu District, Wuxi 214135, China; derry.yang@solmontech.com

**Keywords:** wood, nanoscale alloy, mold resistance, protection efficiency, leaching rate

## Abstract

The mold infection of wood reduces the quality of its surface and potentially endangers human health. One category of the most popular mold inhibitors on the market is water-soluble fungicides. However, easy leaching due to ionic forms is a problem, which reduces the effectiveness of their antimicrobial action, as well as causing environmental pollution. Interestingly, nanometer-sized sterilizing agents present strong permeability and highly fungicidal behavior, and they are not easily leached, due to the unique nanoscale effect, and they have become alternative candidates as marketable anti-mold agents for wood protection. In this study, we first designed and explored a nanoscale alloy (nano silver–copper alloy, nano-AgCu) to treat wood surfaces for mold growth resistance. The results showed that three molds, i.e., *Aspergillus niger*, *Penicillium citrinum* and *Trichoderma viride*, mainly grew on the surface of wood within a depth of 100 μm; and that the nano-AgCu alloy with a particle size of ~15 nm presented improved retention and anti-mold efficiency at a nanomaterial concentration on the wood surface. Its leaching rate increased non-linearly with the increase in nano-AgCu retention and then it showed a gradually decreasing trend. When the concentration reached 1000 mg/L, the nano-AgCu alloy uniformly distributed on the wood surface in a monodispersed state and exhibited a lower retention of 0.342 g/m^2^, with an anti-mold efficiency of more than 75% and a leaching rate of only 7.678%. Such results positioned 1000 mg/L as the toxic threshold concentration of nano-AgCu against the three molds. This study can provide a scientific basis for the analysis of the anti-mold mechanisms of nano-AgCu alloy on wood surfaces and guide the application of nano-metal alloy materials in the field of wood antimicrobials.

## 1. Introduction

Natural wood resources, while being processed into wood products and for applications, consume less energy, emit less carbon dioxide, and store carbon for a longer term, which, accordingly, can be regarded as green and sustainable ecomaterials. Therefore, sufficient and efficient utilization of wood resources can be a carbon-negative technology to combat the global climate challenge [1]. However, wood resources, as biodegradable materials, are naturally susceptible to fungal deterioration, from mold, stain fungi, and wood rot fungi. Such a feature could lead to premature biodegradation of wood, which, in turn, reduces carbon sequestration. In particular, mold can easily reproduce on the surface of wood, even under moderate humidity conditions (~75%), which can further reduce wood quality [2,3,4]. Consequently, exerting an anti-mold treatment on wood not only can be conducive to improving wood service life and promoting the carbon storage accordingly, but it can also contribute to the amelioration of wood quality, protect human health, and promote an alternative utilization of non-renewable resources, which would accordingly benefit the sustainable and environmentally friendly development of modern society.

Presently, the mainstream anti-mold agents for industrial wood applications are water-soluble germicides with the advantages of simple operation procedures, a lower release of volatile organic compounds (VOCs), and a higher sterilizing efficiency [5,6]. However, the release of fungicidal compounds is easily the bottleneck due to their water solubility [7,8]. Nano-sized anti-microbial agents possess the advantages of not being vulnerable due to their particle sizes, which are larger than ions, and that they have durable fungicidal activity due to the slow release of the active ingredients, as well as broad-spectrum sterilization due to additional nano-effects, making them an emerging anti-mold agents for wood [9,10,11,12,13]. Nanosilver, copper, zinc oxide, titanium dioxide, and graphene have been widely explored for application as anti-mold agents for wood protection [12,13,14]. For example, by impregnating bamboo with aqueous nano-silver (14.3 ± 3.6 nm) solution at a concentration of 2 mg/L, 53% of *Aspergillus niger* was inhibited on the surface of bamboo [15]. With southern yellow pine wood, nano-copper and nano-zinc presented a leaching rate of less than 10%, which was greater than that of boron-based fungicide [16]. Wood treated via impregnation with nano-ZnO (100 nm) at a concentration of 0.1%, exhibited effective inhibition against the growth of *Aspergillus niger*, *Trichoderma harzianum* and *Penicillium pinophilum*, and offered a lower leaching rate of only 13% [14]. Nano-silver, after impregnation into Scots pine sapwood at a concentration of 3 g/L, showed a lower leaching rate of less than 15% [17]. ZnO nanoparticles (~200 nm) fabricated on the surface of bamboo using a wet-chemical method offered better resistance against *Aspergillus niger* and *Penicillium citrinum* growth, with infection values of 1.7 and 1.4, respectively [18]. Poplar wood impregnation by nano-TiO_2_-dispersed polyvinyl butyral (PVB) solution, demonstrated an effective inhibition of mold growth, even under dark conditions [19]. These results indicated that nanoscale fungicides could not only inhibit mold growth on the surface of wood-based materials, but also present good leaching resistance. They exhibited different antimicrobial effects and fungicidal mechanisms due to their distinct compositions and structures; thus, combining them could have a cumulative effect, and, accordingly, provide wood with improved fungicidal effects. For example, applying nano-silver loaded TiO_2_ (51.53 nm) to wood-based materials contributed excellent anti-mold effects to the surface against *Aspergillus niger* and *Trichoderma viride* growth, with protection efficiency of 93.33% and 96.67%, respectively [20,21]. Softwood plywood, impregnated with thermally reduced silver nanoparticles supported by titanium dioxide (AgNPs/TiO_2_), demonstrated excellent antifungal effects and showed rare *Aspergillus niger* mold growth on the surface [22]. Nanoscale silver, copper and titanium dioxide particles (40–50 nm) directly brushed onto pine and beech wood surfaces, heavily inhibited the growth of *Aspergillus niger* [23]. Bamboo surface coated with nano-ZnO/graphene exhibited improved mold-resistance, with a grade 2 infection value against *Aspergillus niger* and grade 0 against both *Trichoderma viride* and *Penicillium citrinum*, respectively [24]. All the above studies showed that compositing various types of nanomaterials can have an overlapping effect, further improving anti-mold effects for the wood surfaces.

**Figure 1:**
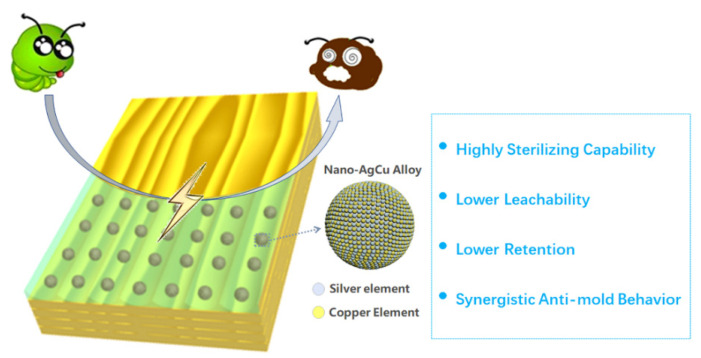
Schematic illustration of the nano silver–copper alloy (nano-AgCu) for mold resistance on the surface of wood.

Consequently, it is reasonable to assume that antifungal metal compounds, formed into a nanoscale alloy, could probably exhibit the multiple effects of components superposition and structure synergy to create an ideal antifungal wood surface. However, such nano-alloy materials have not been reported for use in the prevention of wood mold. In this study, nanoscale silver–copper alloy (nano-AgCu) with a diameter of ~15 nm was designed for loading on a poplar wood surface, with the purpose of reducing the silver dosage and the corresponding costs of the nanomaterial, synergistically providing antifungal effects from the silver and copper elements, as well as their nanoscale effects (Figure 1). This study explored the inhibition effect of nano-AgCu alloy of different retentions on wood surfaces against the *Aspergillus niger*, *Trichoderma viride* and *Penicillium citrinum* growths, and evaluated its leaching resistance. Such results can provide a positive analysis of the antifungal mechanisms of nano-alloys and promote wider applications of a series of nano-alloy materials for the protection of wood against mold.

## 2. Materials and Methods

### 2.1. Materials

The nano-AgCu solution (1500 mg/L) was provided by Solmont Technology Inc. (Wuxi, China). The nano-AgCu with an atomic number ratio of 2:1 was stabilized in aqueous solution using a PVP (polyvinyl pyrrolidone)/PVA (polyvinyl alcohol) mixture. Potato dextrose agar (PDA) (Keshang Biotechnology, Jinan, China) was utilized for the fungi culture. Poplar wood specimens with dimensions of 50 × 20 × 3 mm (L × W × H), were purchased from a local market, without bugs, knots or other defects.

### 2.2. Characterization of Nano-AgCu

The morphology and microstructure of the nano-AgCu alloy and polar wood samples were characterized using field-emission scanning electron microscopy (FESEM) (QUANTA FEG 400, FEI company, Hillsboro, OR, USA) with energy dispersive X-ray spectroscopy (EDX) (GENESIS, EDAX Inc., Los Angeles, CA, USA), high-resolution transmission electron microscopy (HRTEM) using an EDX (JEM-2010F, JEOL, Tokyo, Japan) operated at 300 kV, and atomic force microscopy (AFM) (FM-Nanoview 1000, FSM Precision Instruments, Suzhou, China), and through the use of X-ray diffraction patterns (XRD) (D8 Advance, Bruker, Billerica, MA, USA) using Cu Kα radiation. The alloy particle size distribution was determined by a Laser Nanometer Particle Size Analyzer (Zetasizer-Nano-ZS, Malvern Panalytical, Malvern, UK) and the compositions of the alloy loaded wood were characterized by X-ray photoelectron spectroscopy (XPS) with an ESCALAB 250Xi from Thermo Scientific Corporation (Waltham, MA, USA) using AlKa X-ray radiation.

### 2.3. Moldstrains and Growth Condition

Three different microorganism strains were purchased from the China Forestry Collection Center (CFCC) (Beijing, China) and Agricultural Culture Collection of China (ACCC) (Beijing, China). The fungal strains were *Aspergillus niger* (CFCC 83989), *Penicillam citrinum* (CFCC 89234), and *Trichoderma viride* (ACCC 30595), which represent common molds worldwide, found on wood surfaces. Three fungal strains of mycelia fragments were inoculated on PDA prepared according to the manufacturer’s protocol in Petri dishes (standard 90 mm) at a temperature of 28 °C and relative humidity of 85% for 7 days.

### 2.4. Anti-Mold Test

#### 2.4.1. Mold Resistance of Nano-AgCu on Culture Medium

Aqueous nano-AgCu solution was added to PDA substrate to obtain final concentrations of 3, 30, 300, 500, 700, 1000 mg/L, and a control group (CK) without nano-AgCu in PDA substrate was employed for comparisons. After 7 days of cultivation, a few fragments of mycelia were cut from the edge of the fungal colonies and inoculated in the centers of the nano-AgCu loaded PDA groups and the CK group for 4 weeks at a temperature of 28 °C and relative humidity of 85%. During incubation, the area covered by fungal colonies was measured every week and the inhibition rate (*I*) was calculated over 28 days according to Equation (1).

(1) $$I=\frac{D-d}{D}\times 100\%$$

where *D* is the average diameter of the mycelium on the control plate; *d* is the average diameter of the mycelium on the nano-AgCu plate.

#### 2.4.2. Mold Resistance of Nano-AgCu on Poplar Wood Surface

Anti-mold properties were evaluated according to Chinese Standard GB/T 18261-2013. Briefly, aqueous nano-AgCu solution was prepared at different concentrations of 100, 300, 500, 700, and 1000 mg/L. Poplar wood specimens, weighed before the experiment and followed by sterilization, were immersed in the above-mentioned nano-AgCu solutions, or distilled water (as a control) for 3 min, and were then left to stand overnight and were weighed again. Nano-AgCu retention (*R*) was calculated using Equation (2).

(2) $$R=\frac{\left(m_{2}-m_{1}\right)\times c}{2\times \left(L\times H+L\times W+H\times W\right)}\times 10^{6}$$

where *R* is the retention, g/m^2^; *m*_1_ is the weight of the samples before impregnation, g; *m*_2_ is the weight of the samples after impregnation; *c* is the concentration of the nano-AgCu solution, wt%; *H* is the height of the sample, mm; *L* is the length of the sample, mm; and *W* is the width of the sample, mm.

Then, the wood specimens were placed on the PDA substrates. Notably, the PDA substrate first needed to be coated by fungal strains. Over two sterilized wood rods with dimensions of 70 × 9 × 3 mm (*L × W × H*) were placed on the mycelium-covered PDA substrate, two wood specimens were put separately on the rods separately. After inoculation, the Petri dishes were cultured at 28 °C at a relative humidity of 85% for 4 weeks. The infection value of the mold on the wood specimens is shown in Table 1, and the anti-mold efficiency (i.e., protection efficiency) was obtained using Equation (3).

(3) $$E=\left(1-\frac{D_{1}}{D_{0}}\right)\times 100\%$$

where *E* is the anti-mold efficiency, %; *D*_1_ is the average infection value of the test samples; and *D*_0_ is the average infection value of the control group.

### 2.5. Leaching Test of Nano-AgCu-Treated Wood

Leaching tests were evaluated according to Chinese Standard GB/T 29905-2013. Briefly, six specimens at each concentration were submerged in 180 mL of deionized (DI) water in 500-mL beakers, followed by mild agitation for 14 days. During this procedure, DI water was renewed after 6 h, and at 1-, 2-, 4-, 6-, 8-, 10-, 12-, and 14-day intervals. Meanwhile, leachates were collected. Finally, the total leachate was examined using an ICP method for Ag and Cu contents, and the expression of the leaching rate (μg/mL) was calculated using Equation (4).

(4) $$L=\frac{m_{A}}{m}\times 100\%$$

where *L* is the leaching rate, %; *m*_A_ is the total mass of the Ag and Cu elements in leachates, mg; and *m* is the total mass of the Ag and Cu elements in the polar specimen.

## 3. Results

### 3.1. Characterization of Nano-AgCu Alloy and Its Inhibition Effects against the Three Molds on PDA Plates

**Figure 2:**
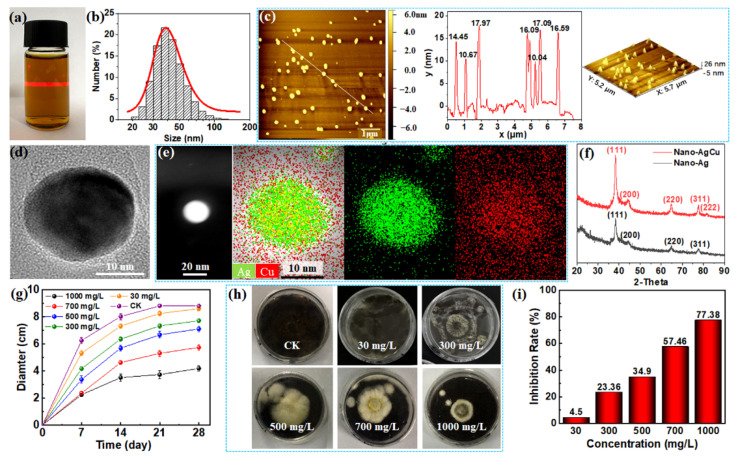
Characterization of nano-AgCu and its inhibition effect against *Aspergillus niger* on the PDA plates. (**a**) The aqueous nano-AgCu suspension passed through by a laser light; (**b**) the size distribution histogram of nano-AgCu particles; (**c**) AFM images of nano-AgCu particles; (**d**,**e**) HRTEM-EDX images of nano-AgCu particle; (**f**) XRD patterns of nano-AgCu and corresponding nano-Ag particles; (**g**) variations of growth diameter of fungus on the PDA plate with growing time at different nano-AgCu concentrations; (**h**) digital photos of fungal growth on the nano-AgCu-treated PDA plate at different concentrations after 28 days; (**i**) inhibition rate of nano-AgCu against fungal growth on the PDA plate at different concentrations.

The aqueous silver–copper suspension had a transparent and light-yellow color and demonstrated a typical Tyndall effect when a laser pointer light passed through it, which featured in a clear beam and indicated that the silver–copper particles were stably suspended at the nanoscale within the solution (Figure 2a). A Laser Nanometer Particle Size Analyzer determined that the particle diameter distribution was in the range of 30–50 nm, with an average size of ~40 nm (Figure 2b). AFM characterization further revealed that the nano-AgCu particles were approximately spherical, with an average diameter of about 15 nm (Figure 2c). TEM observations showed that individual silver–copper particle presented as spherical-like polyhedrons with a diameter of about 15 nm (Figure 2d), which was in agreement with the AFM results. These results revealed that each particle size was smaller than that measured by the particle size analyzer, indicating that the AgCu particles were probably suspended in an aggregate form that was assembled from several particles, but remained at the nanoscale size, and accordingly exhibited the Tyndall effect. TEM-EDX showed that each particle was composed of silver and copper elements with an atom number ratio of 7:3, which was comparable to the feeding ratio of 2:1, representing the thorough reaction and uniform distribution of the two elements in the individual particles (Figure 2e). XRD patterns further revealed that the two metal elements merged into an alloy structure, with crystalline peaks of silver at 38.3° (111), 44.2° (200), 64.6° (220), and 77.8° (311), and copper at 81.8° (222) (Figure 2f). In short, the silver–copper particles presented as a crystalline alloy with a particle size of ~15 nm and were stably dispersed in the aqueous solution.

The aqueous nano-AgCu suspension was blended with the PDA plate at the five concentrations, for further culture of the three molds (*Aspergillus niger*, *Penicillium citrinum* and *Trichoderma viride*). These molds displayed increased mycelial diameters with growth time for each nano-AgCu-treated PDA and the CK group, during the 28-day mold growth term; the mycelial diameter for each nano-AgCu-loaded medium was smaller than that of the control medium. The higher the nano-AgCu concentration of the PDA medium, the smaller the growth diameter of the three molds, which indicated that the nano-AgCu alloy played a certain role in terms of inhibitory function on the three fungi; the higher the concentration, the stronger the inhibitory effect (Figure 2g and Figure S1a,d). Digital photos of the mold growth clearly demonstrated this regularity after the 28-day incubation period (Figure 2h and Figure S1b,e). Generally, nano-Ag or nano-Cu particles sterilize microorganisms by releasing ions; the higher the particle concentration, the greater the number of released ions, and, accordingly, the stronger the sterilization efficiency [25].

In the case of *Aspergillus niger*, the mycelial growth diameter on the nano-AgCu-loaded medium concentration of either 30 or 300 mg/L was close to that of the control group. Meanwhile, the mycelium morphologies were almost similar, and presented as white mycelium covered with mature black spores. On the medium with a higher concentration of nano-AgCu, over 300 mg/L, the mold growth edge exhibited dense, slightly shrinking mycelium, and slight yellow, immature spores, which might have been due to the penetration of nano-AgCu particles into the mycelium in a certain form, resulting in a morphological change of the mold [26]. A similar phenomenon was reported on nano-Ag-loaded medium; that is, with an increasing nano-Ag concentration, the mycelial edges showed a wrinkling morphology [27]. Likewise, *Penicillium citrinum* and *Trichoderma viride* presented similar phenomena to the above-mentioned mold growth (Figure S1b,e). Fungi protect their cell bodies from heavy-metal attack in three ways—extracellular inhibition of the uptake and internalization of metal ions, toxic effect reduction of the entrapped metals via chelation of intracellular biomolecular and metal ions, and by exclusion of metal ions via efflux channels, which thereby mitigate mycelium damage [28]. Consequently, the morphological changes in the above mycelia under high nano-AgCu concentrations originate from their non-specific protection behaviors against the antifungal nanomaterial.

After the 28-day culturing period, the inhibition rates of nano-AgCu against *Aspergillus niger* at concentrations of 300, 500, and 700 mg/L were 23.36%, 34.9% and 57.46%, respectively. Especially, at a concentration of 1000 mg/L, the mycelium growth area was less than a quarter of the total petri dish area, representing an inhibition rate of 77.38% (Figure 2i). The growth area of *Penicillium citrinum* on the nano-AgCu medium was less than half of the total area at each concentration of 300, 500, 700, and 1000 mg/L (Figure S1b), with inhibition efficiency of 60.85%, 92.94%, 95.16%, and 97.99%, respectively (Figure S1c), demonstrating a relatively desirable anti-mold effect. The growth area of *Trichoderma viride* on the nano-AgCu medium was less than half of the total area at concentrations of 700 or 1000 mg/L (Figure S1e), and the inhibition rate reached 53.69% and 88.52%, respectively (Figure S1f), indicating achievement of a comparatively effective anti-mold activity. It was shown that the medium containing 0.1wt% nano-ZnO (~100 nm) inhibited all three molds (*Aspergillus niger*, *Trichoderma harzianum*, and *Penicillium pinophilus*) growths, with each inhibition rate being greater than 50% [14]. Compared to an inhibition rate of over 50%, the nano-AgCu concentration in this study did not exceed 700 mg/L (equal to 0.07 wt%), which was lower than the value of 0.1 wt% reported in the literature, reflecting, to some extent, the anti-mold advantages of nano-AgCu of smaller particle sizes and the alloy morphology.

Briefly, taking an inhibition rate of 50% as a reference, the effective concentrations of nano-AgCu needed to inhibit the growth of *Aspergillus niger*, *Penicillium citrinum*, and *Trichoderma viride* was 700, 300, and 700 mg/L, respectively, which provided a reference for the concentration optimization of nano-AgCu for wood samples.

### 3.2. Anti-Mold Effects of Nano-AgCu against the Three Molds on Wood Surfaces

**Figure 3:**
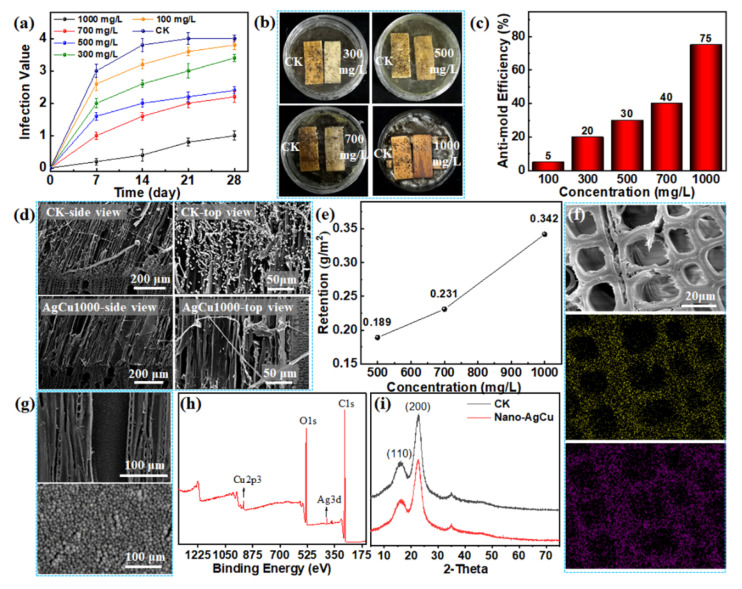
Inhibition effect of the nano-AgCu-treated wood at different concentrations against *Aspergillus niger* growth on polar wood: (**a**) Variations of infection value of different wood surfaces with the mold growth time; (**b**) digital photos of different wood surfaces against mold growth after 28 days; (**c**) variations of anti-mold efficiency of different wood surfaces after 28-day mold growth. (**d**) SEM images of different wood surfaces after fungus infection; (**e**) variations of nano-AgCu retention on wood surface at different concentrations; (**f**) SEM-EDX images of the cross section of nano-AgCu-treated wood at a concentration of 1000 mg/L; (**g**) SEM images of the wood longitudinal surface loaded with nano-AgCu at a concentration of 1000 mg/L; (**h**) XPS spectra of nano-AgCu particles; (**i**) XRD patterns of CK wood and the nano-AgCu (at a concentration of 1000 mg/L) loaded wood surface.

Consulting the above-mentioned effective concentrations of nano-AgCu on PDA substrates, and considering the complex microstructure of wood, concentrations of 300, 500, 700 and 1000 mg/L were employed as the experimental concentrations to explore anti-mold effects on the wood surface. For comparison, a 100 mg/L concentration was also explored. From the results, it was found that the three molds could grow rapidly on the control wood surface, and the mycelium completely covered the whole wood surface after 28 days, indicating that its infection value reached grade 4, according to Table 1. Similarly, each nano-AgCu-treated wood surface was gradually infected with fungi growth. The longer the growth time, the more serious the infection, and the higher the treatment concentration, the slower and weaker the infection (Figure 3a and Figure S2a,e).

For *Aspergillus niger*, the wood surfaces treated with nano-AgCu concentrations less than 1000 mg/L were covered with large bundles of mycelia and resulted in an infection value greater than grade 2, as well as an anti-mold efficiency less than 50% (Figure 3a–c). For the nano-AgCu-treated wood with a concentration of 1000 mg/L, the surface was only infected by small amounts of mycelia, with an infection value of grade 1 and a protection efficiency of 75%, indicating the effective inhibition effects of the nano-AgCu alloy against mold growth under such concentrations.

For *Penicillium citrinum* and *Trichoderma viride*, the nano-AgCu-treated wood surface presented similar regular patterns of mold inhibition to that of *Aspergillus niger* (Figure S2a,e). That is, only the nano-AgCu at a 1000 mg/L concentration inhibited mold growth, represented as wood surface covered by small amounts of mycelia with an anti-mold efficiency of more than 75% (Figure S2b,c,f,g). For this reason, 1000 mg/L was considered to be the toxic threshold concentration of the nano-AgCu against all the three fungi (*Aspergillus niger*, *Penicillium citrinum*, and *Trichoderma viride*) on the wood surfaces. At such a concentration, only small amounts of mycelia, without spores, were observed on the surfaces of the cellular microstructures; there were no mycelium or spores under the surface even at a depth of ~100 μm (Figure 3d and Figure S2d,h). A similar phenomenon of rare mold infection below the wood surface also occurred in the control wood (Figure 3d and Figure S2d,h), where the surface was covered by large amounts of spores and mycelia, revealing that the three molds thoroughly infected the wood surface and accordingly affected the wood quality. However, such behavior did not negatively affect the wood structure, nor its mechanical strength [29]. Consequently, the nano-AgCu particles that were mainly loaded on wood surface could not only inhibit mold growth, but also reduce the dosage of the antimicrobial agent. In addition, nano-AgCu retention on the wood surface was only 0.342 g/m^2^ when the concentration was 1000 mg/L (Figure 3e), which effectively inhibited mold growth. The literature has reported that, when copper ions released from micronized copper oxide exceeded 1.0 g/m^2^, they could effectively inhibit the mold growth [25]. Normally, the antimicrobial activity of nanomaterials increases with a decrease in their particle sizes [30]. Therefore, compared to micro-copper oxide, the nano-AgCu alloy demonstrated a greater antifungal effect, with the advantages of having smaller particle size and a lower dosage.

After nano-AgCu at a 1000 mg/L concentration was deposited on the wood surface, the porous lumen structure on the cross-section and pit apertures on the longitudinal cell wall surface were clearly visible, indicating the retention of the original microstructure of the wood (Figure 3f,g). SEM-EDX showed that the silver and copper elements were fully and evenly spread out on the cell wall surface, indicating the uniform distribution of the nano-AgCu alloy (Figure 3g). The particle size on the cell wall surface was comparable to that of the original nano-AgCu alloy (Figure 3f), demonstrating that the nano-AgCu particles were monodispersed on the cell wall without obvious aggregation, and the cell lumen-pit channels were unblocked due to the small particle sizes, which accordingly ensured the smooth penetration of nanoparticles into the microscopic channels. XPS also proved the deposition of nano-AgCu particles on the wood surfaces; however, the silver and copper element peaks were quite weak, probably owing to their lower weight loading (Figure 3h), which was proved by the lower retention of 0.342 g/m^2^. XRD results revealed that the nano-AgCu-treated wood presented a similar crystalline structure to the control wood, which further proved the lower retention (Figure 3i).

In short, the nano-AgCu particles remained in a monodispersed state after loading on wood surfaces at a concentration of 1000 mg/L, which guaranteed its antifungal function to effectively inhibit fungi growth [31].

Generally, nanometals like nano-silver or nano-copper mainly sterilize microorganisms via ion release, which can penetrate their cell wall and interact with sulfhydryl and phosphate groups, resulting in the death of the microorganisms [32]. The ion-releasing capability of nanometals is directly influenced by this, as well as the physicochemical environment during applications, and factors such as particle size, ambient pH, temperature, humidity, and oxidation atmosphere. The conditions of small particle size, an acidic environment, and the oxidation environment is beneficial for ion release [17,25,33,34]. The small particle size of the nano-AgCu alloy (only about 15 nm), the weak acidic environment (pH ≈ 4.0–6.5), and the oxidative functional groups of wood can promote ion release, and the nano-alloy thereby exhibits a strong antifungal effect. Commonly, the more nanoparticles there are, the greater the number of ions released, and the greater the antifungal efficiency produced. When the nano-AgCu alloy is loaded on wood surfaces in an aqueous suspension, the retention increased with the concentrations (Figure 3e), and the anti-mold efficiency increased accordingly (Figure 3c and Figure S2).

**Figure 4:**
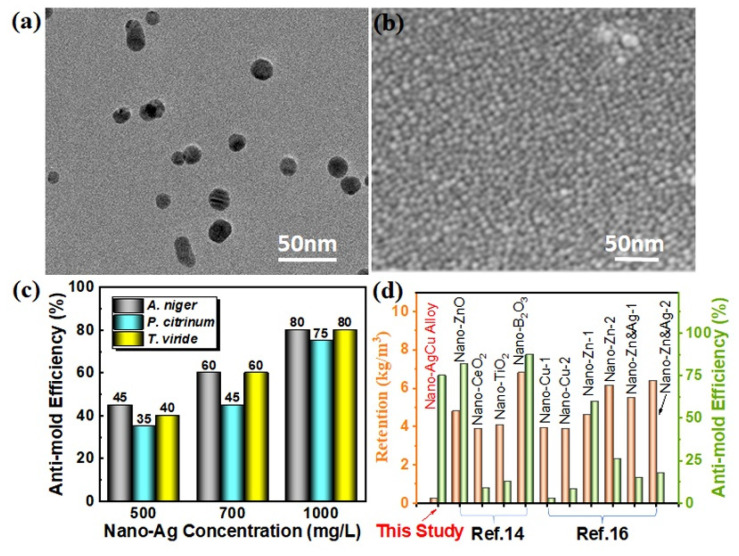
Comparison of the inhibition effect of nano-AgCu with nano-Ag against three molds to evaluate the potential synergy effect of Ag and Cu elements: (**a**) TEM image of nano-Ag particles; (**b**) SEM image of nano-Ag particles loaded on wood surface at a concentration of 1000 mg/L; (**c**) variations of the anti-mold efficiency of nano-Ag against three molds with the particle concentration; (**d**) comparison of the anti-mold efficiency and retention of nano-AgCu with other reported anti-mold nanoparticles in the literature. (Terzi et al., 2016), Copyright 2016, Elsevier; (Kartal et al., 2009), Copyright 2009, Elsevier.3.3. Leachability of Nano-AgCu Particles on Wood Surface.

To further differentiate the alloy and single metal functions for resistance to fungi growth, nano-Ag with a similar particle size to that of the nano-AgCu was employed to treat wood surfaces. The nano-Ag diameter was about 15 nm (Figure 4a), and the particles were uniformly monodispersed on wood surface (Figure 4b) at each concentration (500, 700, and 1000 mg/L). The resulting nano-Ag-treated wood presented a similar regular pattern of anti-mold efficiency to the above-mentioned nano-AgCu. In other words, the anti-mold efficiency of nano-Ag against all three molds increased with the concentration imposed on the wood surface, which was consistent with that of nano-AgCu (Figure 3c and Figure S2c,g).

Commonly, silver ions exhibit better antifungal activity than copper ions [17]. When both nano-Ag and nano-AgCu, with similar particle sizes, are separately to wood surfaces under the same conditions, it is reasonable to assume that their ion-release abilities are comparable. Therefore, taking only ion-release way into account, nano-Ag should present a higher antifungal capability than nano-AgCu alloy. Comparing the antifungal activities of the two nanomaterials at the same concentrations, it was found that the anti-mold effects of nano-Ag, against *Aspergillus niger* and *Trichoderma viride*, were higher than that of nano-AgCu (Figure 4c), respectively, which was consistent with the above deduction. However, for *Penicillium citrinum*, the anti-mold efficiency of nano-AgCu was better than that of nano-Ag (Figure 4c); in other words, a combination of silver and copper elements in the alloy morphology realized a better inhibition of fungal growth, which indicates a certain degree of synergistic effect of the nanoalloy against mold.

Additionally, compared to the retention and anti-mold efficiencies of other antifungal nanomaterials reported in the literature, it was found that the nano-AgCu-treated wood obtained almost the highest anti-mold efficiency with the lowest retention (Figure 4d), representing an excellent antimicrobial activity.

**Figure 5:**
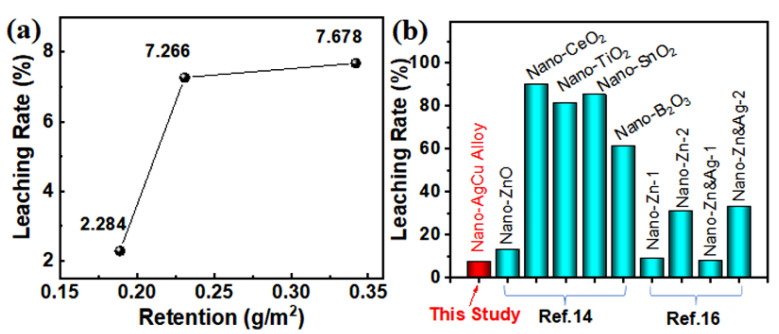
Leachability of nano-AgCu particles on poplar wood surface: (**a**) Variations of leaching rate of nano-AgCu with its retention; (**b**) comparison of the leachability of nano-AgCu alloy with other reported anti-mold nanoparticles in the literature. (Terzi et al., 2016), Copyright 2016, Elsevier; (Kartal et al., 2009), Copyright 2009, Elsevier.

Antimicrobial materials commonly have higher leachability due to their waterborne behaviors, which reduces antifungal activity during applications. Consequently, improving their anti-leaching capability favors a durable antifungal effect [35]. Generally, antimicrobial materials at the nanoscale possess a huge specific surface area and large numbers of active sites, which can promote interactions of the nanomaterials with functional groups on cell wall components, and thus reduce its leachability [17,25]. Moreover, wood can load abundant nanomaterials due to its high porosity, and the porous structure assists the anti-leaching capability of antifungal nanomaterials [8,12]. Therefore, there is a non-linear positive relationship between leaching resistance and retention of the antifungal nanomaterial during their deposition on the cell wall surface prior to reaching monolayer saturation. After that, more agglomeration would occur among the nanomaterials due to their van der Waals forces, thus reducing the adhesion of the outer nanomaterials to the wood cell walls, which further promotes the leaching trend of the nanomaterial with an increased slope. Figure 5a shows that the leaching rate of the nano-AgCu on wood surfaces increased non-linearly with retention, and the leaching trend gradually decreased after that. The nano-alloy retention reached 0.342 g/m^2^ at a concentration of 1000 mg/L, while the leaching rate was only 7.678%, which completely indicates that the nanomaterial, under such retention, mainly deposited on the cell wall surface without unsaturation. This deduced conclusion was indirectly proved by the nano-AgCu distribution on the cell wall surface that is presented in Figure 3g. Such result guarantees the effective performance of its anti-mold activity, which is in accordance with that reflected in Figure 4d.

Previous studies reported that the southern yellow pine sapwood was vacuum-impregnated by nano-ZnO (30 and 70 nm) at three concentrations (1%, 2.5%, 5%) and soluble ZnSO_4_, respectively, and the chemical leaching rate was also tested. It was found that the leaching rate of the nano-ZnO-treated wood was less than 4%, while the waterborne ZnSO_4_-treated wood reached 13–25%, and the nano-ZnO (30 nm) was essentially non-leachable at a treatment concentration of 5% [36]. These results indicated that nanomaterials have better leaching resistance, and their leaching rate decreased with a decrease in particle sizes, and the retention increased with the concentration, which was consistent with the results shown in this study. Furthermore, it was found that, compared with other antimicrobial nanomaterials reported in the literature, nano-AgCu-treated wood at a concentration of 1000 mg/L in this study presented the lowest leaching rate (Figure 5b), which benefited the antifungal function.

In conclusion, the nano-AgCu alloy with quite tiny particle sizes and a lower retention was uniformly loaded on the wood cell wall surface, and it imparted satisfactory antifungal effects and resulted in a desirable anti-leaching rate for the treated wood.

## 4. Conclusions

- The retention and antimicrobial effectiveness of the nano-AgCu alloy on wood surfaces increased with the nanomaterial concentration, and the leaching rate increased non-linearly with retention;
- The combination of the advantages of the nano-AgCu alloy with quite small particle sizes (~15 nm), good distribution on wood surfaces, and the synergistic effect of the two elements promoted the achievement of a strong anti-mold effect (inhibition efficiency >75%) at lower retentions (0.342 g/m^2^); and
- The toxic threshold concentration of the nano-AgCu alloy against the three molds was 1000 mg/L, and the leaching rate only reached 7.678% due to strong interactions between the alloy and wood cell walls.

## Figures and Tables

**Table 1:** Standard method for determining infection values.

Infection Value	Mold Coverage
0	No fungal growth on the sample surface
1	Surface infection area <1/4
2	Surface infection area 1/4~1/2
3	Surface infection area 1/2~3/4
4	Surface infection area >3/4

## Data Availability

Data presented in this article are available at request from the corresponding author.

## Supplementary Materials

The following supporting information can be downloaded at: https://www.mdpi.com/article/10.3390/nano12071192/s1, Figure S1: Characterization of nano-AgCu and its inhibition effect against *Penicillium citrinum* and *Trichoderma viride* on PDA plate; Figure S2: Inhibition effect of the nano-AgCu with different concentrations against the *Penicillium citrinum* and *Trichoderma viride* growth on polar wood.
