# Supplementary material for: Nano-AgCu Alloy on Wood Surface for Mold Resistance
Source: Nanomaterials (Basel). 2022 Apr 2;12(7):1192. doi: 10.3390/nano12071192 (PMC9002835; doi:10.3390/nano12071192)
Supplement: Supplementary file 1 [file nanomaterials-12-01192-s001.zip › nanomaterials-1615874-supplementary.pdf]

nanomaterials

Supporting Information

Nano-AgCu Alloy on Wood Surface for Mold Resistance

Yanran Qi [1,†], Xiaohan Dai [1,†], Lianxiang Wei [1], Hongxue Luo [1], Yiliang Liu [1], Xiaoying Dong [1,*], Dequan Yang [2] and Yongfeng Li [1,*]

[1] State Forestry and Grassland Administration Key Laboratory of Silviculture in Down-stream Areas of the Yellow River, College of Forestry, Shandong Agricultural University, Taian 271018, China; qyran1994@163.com (Y.Q.); dxiaohan0315@126.com (Xiaohan Dai); 15662006529@163.com (L.W.); luohongxue1997@163.com (H.L.); liuyiliang0207@163.com (Yiliang Liu); xydong@sdau.edu.cn (X.D.)

[2] Solmont Technology Wuxi Co., Ltd., 228 Linghu Blvd., Tian'an Tech Park, A1-602, Xinwu District, Wuxi, 214135, China; derry.yang@solmontech.com

* Correspondence: xydong@sdau.edu.cn (X.D.); Tel.: +86 538 8240610 (X.D.); yfli@sdau.edu.cn Yongfeng Li; Tel.: +86 538 8240610 (Y.L.)

† Yanran Qi and Xiaohan Dai contribute equally to this work.

Citation: Qi, Y.; Dai, X.; Wei, L.; Luo, H.; Liu, Y.; Dong, X.; Yang, D.; Li, Y. Nano-AgCu Alloy on Wood Surface for Mold Resistance. *Nanomaterials* **2022**, *12*, 1192. https://doi.org/10.3390/nano12071192

Academic Editors: Kamil Kaminski and Miguel Gama

Received: 12 February 2022
Accepted: 24 March 2022
Published: 2 April 2022

Publisher's Note: MDPI stays neutral with regard to jurisdictional claims in published maps and institutional affiliations.

Copyright: © 2022 by the authors. Submitted for possible open access publication under the terms and conditions of the Creative Commons Attribution (CC BY) license (https://creativecommons.org/licenses/by/4.0/).

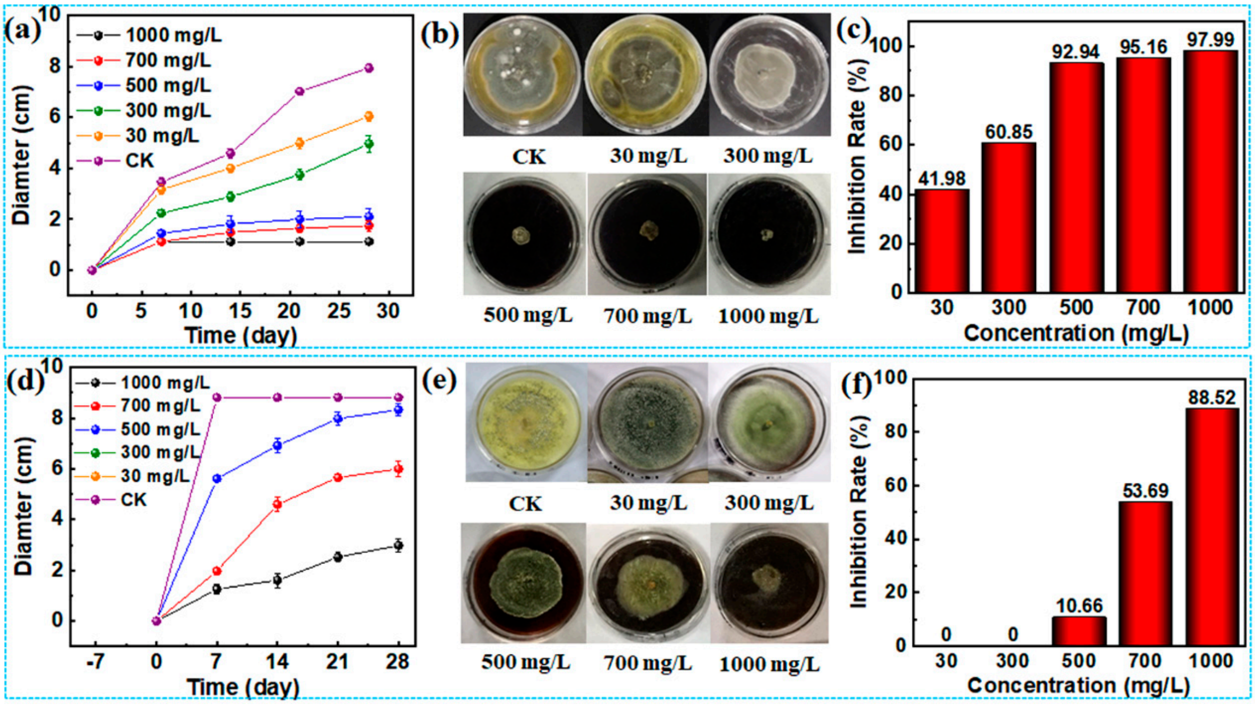

Figure S1. Characterization of nano-AgCu and its inhibition effect against *Penicillium citrinum* and Trichoderma viride on PDA plate. (a) Variations of growth diameter of the *Penicillium citrinum* on PDA plate with growing time under different nano-AgCu concentrations; (b) Digital photos of the *Penicillium citrinum* grown on nano-AgCu treated PDA plate with different concentrations after 28 days; (c) Inhibition rate of the nano-AgCu against the *Penicillium citrinum* growth on PDA plate under different concentrations; (d) Variations of growth diameter of the *Trichoderma viride* on PDA plate with growing time under different nano-AgCu concentrations; (e) Digital photos of the *Trichoderma viride* grown on nano-AgCu treated PDA plate with different concentrations after 28 days; (f) Inhibition rate of the nano-AgCu against the *Trichoderma viride* growth on PDA plate under different concentrations.

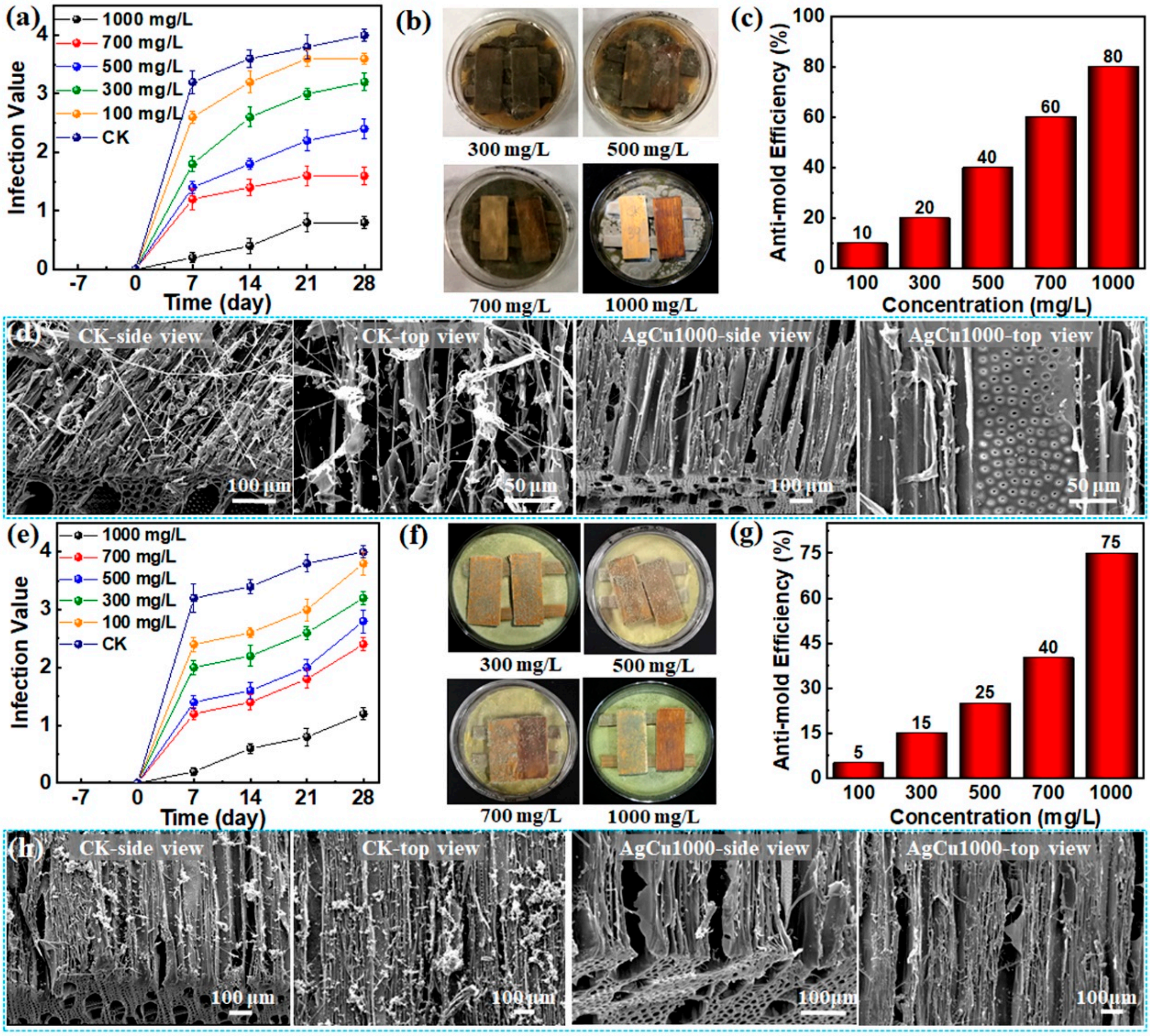

Figure S2. Inhibition effect of the nano-AgCu with different concentrations against the *Penicillium citrinum* and *Trichoderma viride* growth on polar wood: (a) Variations of infection value of different wood surfaces with the *Penicillium citrinum* growth time; (b) Digital photos of different wood surfaces against the *Penicillium citrinum* growth after 28 days; (c) Variations of anti-mold efficiency of different wood surfaces after 28-day growth of *Penicillium citrinum*; (d) SEM images of different wood surfaces after the *Penicillium citrinum* infection; (e) Variations of infection value of different wood surfaces with the *Trichoderma viride* growth time; (f) Digital photos of different wood surfaces against the *Trichoderma viride* growth after 28 days; (g) Variations of anti-mold efficiency of different wood surfaces after 28-day growth of *Trichoderma viride*; (h) SEM images of different wood surfaces after the *Trichoderma viride* infection.
